# FLAMES overlaying anti-N-methyl-D-aspartate receptor encephalitis: a case report and literature review

**DOI:** 10.1186/s12883-024-03617-z

**Published:** 2024-04-25

**Authors:** Rimei Zhong, Xiongjin Chen, Feng Liao, Zhijun Lin, Zhijian Zhang, Yusen Chen, Lili Cui

**Affiliations:** 1 Guangdong Key Laboratory of Age-Related Cardiac and Cerebral Diseases, Affiliated Hospital of Guangdong Medical University, Zhanjiang, China; 2 Department of Neurology, Affiliated Hospital of Guangdong Medical University, Zhanjiang, 524000 China

**Keywords:** FLAMES, Anti-N-methyl-D-aspartate receptor, Autoimmune encephalitis, Seizures

## Abstract

**Background:**

In recent years, simultaneous or sequential occurrence of MOG antibody disease and anti-NMDAR encephalitis in the same patient has been reported with increasing frequency. Scholars refer to the overlapping occurrence of these two disorders as MOG antibody disease and anti-NMDAR encephalitis overlap syndrome (MNOS). Cortical T2-weighted fluid-attenuated inversion recovery (FLAIR) -hyperintense lesions in anti-MOG-associated encephalitis with seizures (FLAMES) is a rare clinical phenotype of MOGAD in which cortical FLAIR high-signal lesions are unilateral, with little spread to the cortex and meninges bilaterally. Although cases of FLAMES have been consistently reported. However, to our knowledge, such cases of FLAMES combined with NMDARE are rare.

**Case presentation:**

Here, we describe a case of FLAMES combined with anti-NMDARE. The patient was a young male, 29 years old, admitted to our hospital with isolated seizures, whose MRI showed unilateral thalamic and bilateral frontal and parietal leptomeningeal involvement. Since we were unaware of the possibility of bilateral meningo-cortical MOGAD manifestations, the case was initially diagnosed as viral encephalitis and was given antiviral therapy. The diagnosis was not clarified until anti-NMDAR-IgG and MOG-IgG positivity was detected in the cerebrospinal fluid and serum. The patient was then treated with high-dose corticosteroids and his symptoms responded well to the steroids. Therefore, this case expands the clinical spectrum of MNOS overlap syndrome. In addition, we describe the clinical features of MNOS by summarizing the existing literature and exploring the possible mechanisms of its immune response.

**Conclusions:**

Our case serves as a reminder to clinicians that when patients present with atypical clinical manifestations such as seizures, consideration should be given to MNOS and conduct testing for various relevant autoantibodies (including MOG abs) and viruses in both serum and cerebrospinal fluid, as it is easy to misdiagnose the disease as other CNS diseases, such as viral meningoencephalitis. This syndrome exhibits a high responsiveness to steroids, highlighting the critical importance of recognizing the clinical and neuroimaging features of this overlap syndrome for prompt diagnosis and treatment. Furthermore, it enriches the disease spectrum of MNOS.

**Supplementary Information:**

The online version contains supplementary material available at 10.1186/s12883-024-03617-z.

## Background

Anti-N-methyl-D-aspartate receptor (NMDAR) encephalitis (NMDARE) is the most frequently occurring type of autoimmune encephalitis (AE), and its mechanism is primarily related to IgG antibodies(abs) on the nerve surface against the NMDAR GluN1 subunit [1, 2], 78–86% of patients showed a rapid cognitive decline, psychosis and seizures [3]. Myelinating Oligodendrocyte Glycoprotein Antibody-Associated Disease (MOGAD) is an inflammatory demyelinating disease most commonly characterized by clinical manifestations of optic neuritis (ON), myelitis, and acute disseminated encephalomyelitis(ADEM), whereas cortical encephalitis with seizures is rare [4]. Cortical T2-weighted fluid-attenuated inversion recovery (FLAIR) -hyperintense lesions in anti-MOG-associated encephalitis with seizures (FLAMES) is a rare clinical phenotype of MOGAD, which was initially reported as unilateral cortical encephalitis, but it can also manifest as bilateral cortical lesions with or without leptomeningeal involvement or even isolated unilateral leptomeningeal enhancement [5, 6]. The typical clinical presentation is characterized by fever, headache, seizures, and cortical symptoms corresponding to the site of the lesion [6]. In recent years, the coexistence of anti-NMDAR-IgG and MOG-IgG has been identified in some rare reported cases [3, 7–16], and this simultaneous or sequential occurrence of MOGAD and NMDARE in the same patient has been referred to as this MOGAD and NMDARE overlap syndrome (MNOS) [17]. The underlying mechanisms and clinical features of this antibody-immunological coexistence are currently unclear. Even though FLAMES are being detected with increasing frequency, cases of FLAMES coexisting with NMDARE are rare. Here, we report a case of FLAMES combined with NMDARE, and by summarizing the available literature, we describe the clinical features of MNSO and explore the possible mechanisms of its immune response.

## Case presentation

**Fig. 1:**
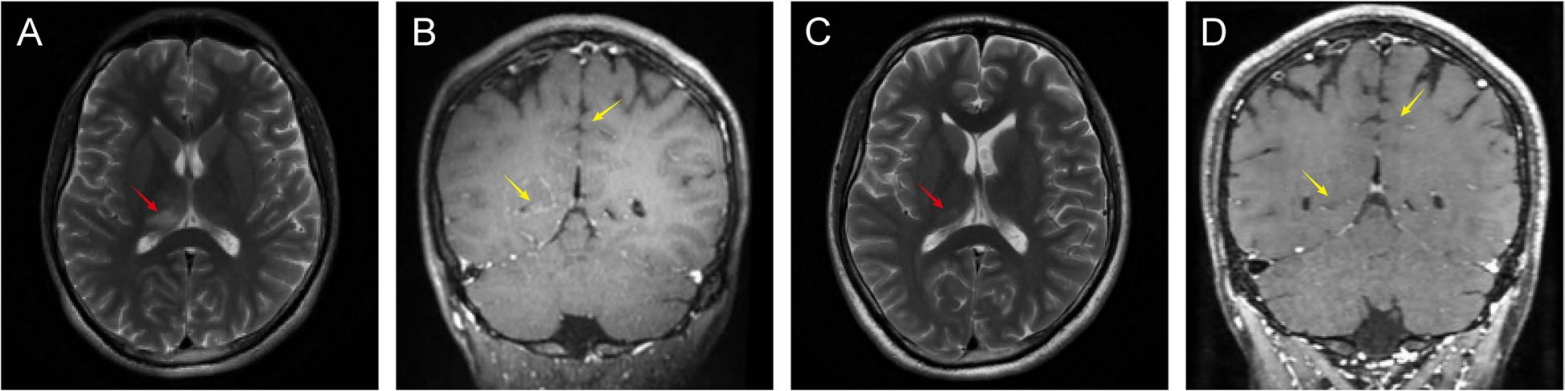
Brain MRI performance. **A**-**B** The MRI data of the patient 6 days after the symptom onset showed hyperintensity of the right thalamus on T2-weighted imaging (red arrows), and the pia mater is strengthened( yellow arrows). **C**-**D** Brain MRI scan performed 168 days after the initial symptom onset depicted a significant improvement in the imaging abnormality

**Fig. 2:**
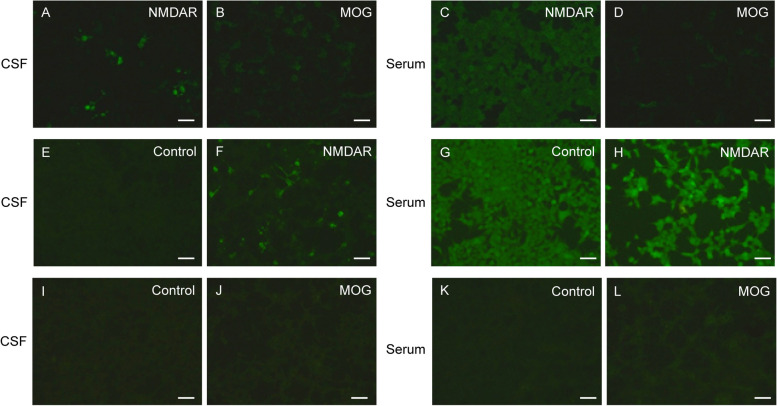
Examination of NMDAR and MOG antibodies in cerebrospinal fluid and serum of our patient. Positive antibodies were confirmed by HEK293 transfected cells via the indirect immunofluorescence method (fixed cell-based assay). MOG-ab uses full-length human MOG to detect MOG-IgG (secondary antibody: IgG Fc). **A**-**D** CSF NMDAR and MOG antibodies were positive (titer 1:10 and 1:3.2, respectively) while serum NMDAR and MOG antibodies were positive (titer 1:10 and 1:32, respectively) on day 8. **E**-**L** Recheck the antibody on day 168. CSF (**E**, **F**) and serum (**G**, **H**) NMDAR antibodies were positive (titer 1:1 and 1:10, respectively) while CSF (**I**, **J**) and serum (**K**, **L**) MOG antibodies were negative and positive (titer 1:10), respectively. All images have a scale of 40X

**Fig. 3:**
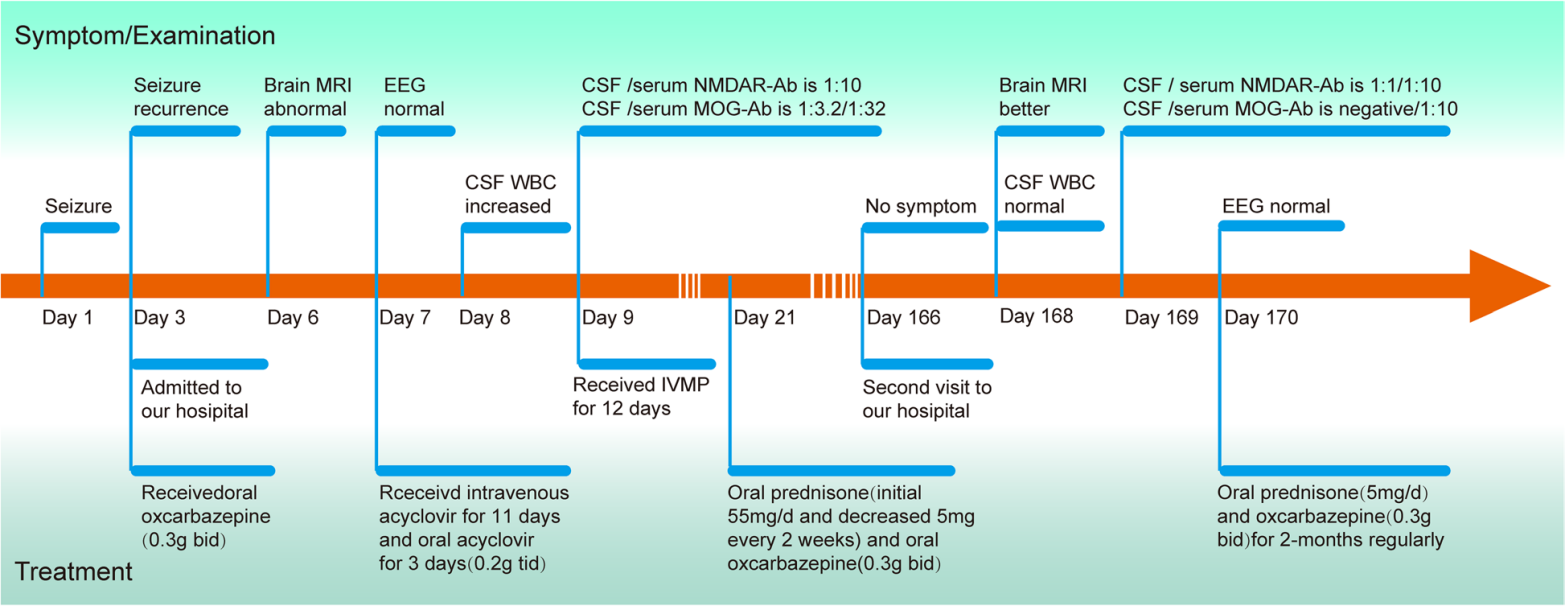
Timeline with clinical manifestation, treatment progression, and diagnosis time. EEG: Electroencephalograph; MRI: Magnetic Resonance Imaging; IVMP: Intravenous methylprednisolone; Abs, antibodies; WBC: white blood cell; CSF: cerebrospinal fluid; NMDAR: Anti-N-methyl-D-aspartate receptor; MOG: Myelin oligodendrocyte glycoprotein

A formerly healthy 29-year-old Chinese male was admitted to our hospital (Affiliated Hospital of Guangdong Medical University, Zhanjiang, China) with isolated seizures, presenting with flexion of the left upper extremity and extension of both lower extremities, foaming at the mouth, rolling of the eyes, unconsciousness, without fever, headache, diplopia, blurred vision, or unsteady gait. The whole episode lasted about 5 min, not progressing to status epilepticus, with a total of 2 episodes. To control seizures, oxcarbazepine is given on the day of admission. On day 6 of the course of the disease, his brain Magnetic Resonance Imaging (MRI) showed lesions in the right thalamus, along with soft meningeal enhancement in the bilateral top frontal and right occipital areas (Fig. 1A-B), and the results considered meningoencephalitis as a high possibility (more inclined to viral infection). The patient is positive for CMV abs IgG, Rubella virus antibody (ab) IgG positive, and Toxoplasma ab IgG positive. However, he denied a history of antecedent infections. The initial diagnosis was viral meningoencephalitis, followed by empiric treatment with acyclovir 0.5 g Q8h. However, according to the patient's clinical symptoms and brain MRI results, there is no exclusionary autoimmune encephalitis, central nervous system (CNS) demyelinating disease, or other diseases. To determine the cause, we performed a lumbar puncture. The opening pressure of cerebrospinal fluid (CSF) was normal, and CSF analysis showed leukocytosis of 20 × 10^6/L, 75% monocytes, normal protein, glucose, and chlorine, suggesting mild inflammation. There are no bacteria, fungi, acid-fast bacilli, cryptococcus, or viruses in the CSF. EEG results are normal. No tumor was detected on CT of the chest, abdomen, and pelvis. In addition, the report showed that autoantibodies against AE and demyelinating diseases were positive in CSF and serum (Fig. 2A-D), the anti-NMDAR abs titers were (1:10) and (1:10) in CSF and serum, MOG-ab in CSF and serum were (1:3.2) and (1:32), respectively. AQP4 ab, anti-MBP ab, anti-GABA B receptor ab, anti-mGLUR5 ab, and anti-CASPR2 ab were all negative. We finally diagnosed the patient with MOGAD and NMDARE overlap syndrome (MNOS). He responded well to immunomodulatory therapy (methylprednisolone, 1000 mg, × 3 d; later reduced to 500 mg, × 2 d, 240 mg × 3 d, 120 mg × 3 d intravenously), and did not have seizures during the hospital. He continued to take oral prednisone acetate tablets in discharge (55 mg, qd, minus 1 tablet every 2 weeks) and oxcarbazepine (0.3 g bid) outside the hospital. The patient was admitted to the hospital for re-examination, He had a good prognosis, no recurrence of seizures, no neurological sequelae, and no impact on his daily living activities. Surprisingly, on day 168 of the course of the disease, his brain MRI showed that the right thalamus lesion was a flaky abnormal signal shadow, which was significantly absorbed and reduced compared with before (Fig. 1C-D). The degree of pia mater strengthening of the top of the bilateral frontal and right occipital is significantly reduced compared with the previous one. The results of autoantibodies against AE and demyelinating diseases in CSF and serum showed that anti-MOG abs were negative in CSF and serum (1:10), CSF and serum anti-NMDAR abs were (1:1) and (1:10), respectively (Fig. 2E-L). Residual CSF analysis and EEG were normal. So far, there has been no recurrence at follow-up. A graphical summary (Fig. 3) shows the entire course of the disease, diagnosis, and treatment.

## Discussion and conclusions

Several recent cases have reported that MOG abs and NMDAR abs can be present simultaneously [3, 7–16]. However, the potential mechanisms of MOG-IgG and NMDAR-IgG immune coexistence and their co-incidence are unclear. By reporting a rare case and reviewing the literature, this article describes the clinical features of MNOS and explores the possible mechanisms of its immune response.

### Seizures are an atypical symptom of MNOS syndrome, and MNOS should exclude other viral and autoimmune diseases

Seizures are a common manifestation of AE [18], occurring in the early stages of approximately 70 percent of patients with NMDARE [19]. However, MOG-IgG has recently been associated with cortical encephalitis and seizures [20]. Seizures can arise in isolation in cases of single-antibody disorders and are also more frequently observed in male patients [1, 21, 22]. It has been reported in some rare cases that anti-NMDAR encephalitis and demyelinating diseases can coexist in the same patient while developing serological markers of autoimmune diseases and demyelinating (MOG abs), while we summarize the cases of seizures present in the course of the MNOS [3, 7–16] in (see Supplementary file 1). In a cohort of 533 patients, Fan et al. identified the presence of both anti-NMDAR abs and anti-MOG abs in 12% of patients and collectively termed it MOGAD and NMDARE overlap syndrome (MNOS) [17]. The age of onset of these syndromes tends to be young, with more males than females, fewer cases of comorbid tumors, and more frequent recurrent disease courses [7, 17, 23]. More notably, the clinical presentation of MNOS is often atypical, with first-onset or single relapsing course presenting with coexistence of anti-NMDAR encephalitis and CNS demyelinating disease, or only anti-NMDAR encephalitis or CNS demyelinating disease alone (see Supplementary file 1). This makes the diagnosis of clinical cases of overlapping MOGAD and NMDARE often difficult by presenting more complex symptoms. Typical NMDARE is readily distinguishable from MOG antibody-associated demyelinating disease. It is difficult to identify patients with anti-NMDAR encephalitis with a combination of positive MOG abs when the clinical manifestations of the two overlap. Studies have shown that patients who are positive for MOG abs and anti-NMDAR abs most often have encephalopathy and seizures [24, 25], and the clinical presentation of MNOS seems to be more similar to NMDARE than to MOGAD [25]. The difference is that the clinical presentation in the present study seems to be more similar to MOGAD than to NMDARE, because of its radiologic features. In conjunction with Supplementary file 1, we learned that seizures in double antibody-positive cases can occur either during the initial or recurrent course of the disease and are often accompanied by other symptoms, whereas double antibody-positive cases with isolated seizures are rarely reported. This case highlights the diagnostic difficulties of the overlap between MOG antibody-associated disease and anti-NMDAR encephalitis, which can be misdiagnosed as viral meningoencephalitis at an early stage due to atypical clinical features. In addition to this, cases of HHV-7 encephalitis combined with MOG and NMDAR ab positivity, and overlap of GFAP-IgG ab and NMDAR ab manifesting seizures have been reported [26, 27]. Therefore, in patients with seizures, such as the cases cited in this study, simultaneous measurement of autoimmune encephalitis abs and MOG abs should be performed at the earliest possible time, as distinct from other viral and autoimmune diseases.

### MRI of MNOS overlaps more with MOGAD, and is meningeal enhancement a clue to the coexistence of dual antibody positivity?

The MRI of patients with MNOS overlapped more with MOGAD [25, 28], and had a higher proportion of supratentorial lesions [17, 28], with frequent involvement of the cortex, subcortical white matter, and basal ganglia regions. The results of a study of 58 double antibody-positive patients also showed that the cortex was the most common MRI change and found that the leptomeninges were more likely to be violated in patients with MNOS [25]. In addition to this, they found that anti-NMDAR abs seemed to contribute to cortical destruction and seizures in MOG-IgG patients. Specific MRI abnormalities are associated with acute symptomatic seizures in MOGAD [22], mainly including unilateral/bilateral cortical T2/fluid attenuation inversion recovery (FLAIR) high-signal lesions, leptomeningeal enhancement, etc., with unilateral cortical T2/FLAIR high-signal lesions (FLAMES) being more likely to have seizures. FLAMES has different radiologic manifestations and may show bilateral cortical and meningeal lesions that are different from the common manifestations [6]. In addition to this, isolated seizures are one of the representative manifestations of FLAMES [29]. Therefore, we believe that the present study is consistent with the diagnosis of FLAMES. Although the role of MOG abs in MOG antibody-associated with seizures is questionable [30] and may not even be involved in the cortical lesion phenotype [31], the increasing number of related cases reported makes the disease phenotype impossible to ignore. FLAMES is more likely to be misdiagnosed as other CNS diseases such as viral meningitis, subarachnoid hemorrhage, or CNS vasculitis [5, 6]. Our case was diagnosed as viral encephalitis initially, as well, because of the lack of awareness of this bilateral meningeal MOGAD disease phenotype. Therefore, increased knowledge and understanding of this disease phenotype is critical to facilitate timely diagnosis. Leptomeningeal enhancement has been proposed as a possible clue to the coexistence of MOG and anti-NMDAR abs [24], but we found that leptomeningeal enhancement was rare in patients with MNOS (see Supplementary file 1). Further studies are needed to confirm this claim because of the limited and small number of cases. This study expands the disease spectrum of MNOS, but its seizure-associated MRI needs further studies to be discovered.

### The exact mechanism of the positive formation of double antibodies is currently unclear

**Fig. 4:**
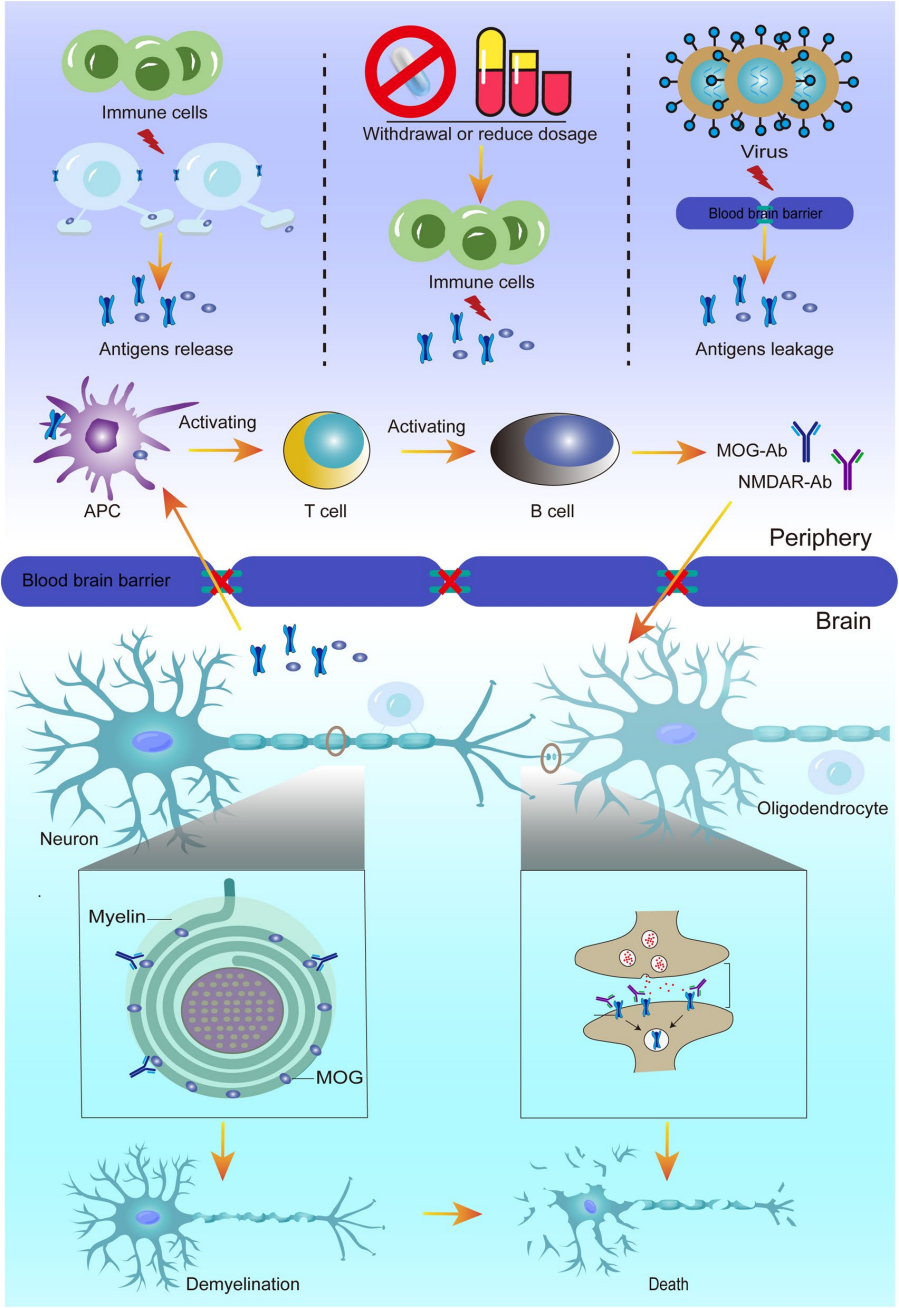
Ilustrates the potential pathogenesis of anti-NMDAR and MOG biantibody-positive autoimmune encephalitis. When immune cells directly attack oligodendrocytes, immune reconstitution resulting from withdrawal or dosage reduction as well as virus destroy the blood–brain barrier, NMDAR and MOG antigens are exposed and presented by antigen presenting cells, followed by T and B cell activation, leading to the production of anti-NMDAR and MOG double antibodies, resulting in demyelination and NMDAR invagination degradation, and eventually neuronal death

The reason for the overlap of these two antibodies is currently unclear and may be related to the following factors. Firstly, the similar or identical proteins expressed on different cells may act as a common antigen and have cross-immune effects with the same ab. Research has confirmed that the surface of oligodendrocytes contains both functional NMDAR and MOG antigens(ags) [32, 33]. Under autoimmunity targeting oligodendrocytes, these two antigens may be simultaneously or sequentially attacked by immune cells and produce anti-NMDAR and anti-MOG antibodies at the same location [8, 34]. Secondly, when the dosage is reduced or immunotherapy is stopped, the previously suppressed immune system recovers and reconstructs, which may lead to immune cells attacking their antigens and producing overlapping antibodies [35, 36]. Literature reports show that patients initially diagnosed with anti-NMDAR encephalitis or MOG antibody disease may respectively experience demyelination pathology or clinical manifestations including mental and behavioral abnormalities, and memory loss during hormone reduction, followed by positive MOG and NMDAR abs [34, 37], suggesting that immune reconstitution is related to MNOS. Finally, some case reports have shown that virus infection may be associated to the production of double antibodies in MNOS patients [7, 38]. Wang et al. believed that virus invasion cause damage to the blood–brain barrier exposed to MOG ags and produced corresponding antibodies, leading to increased damage to the blood–brain barrier, NMDAR ags exposure, and NMDAR abs production [28]. This is similar to the mechanism of virus induced MOG-AD hypothesis proposed by Cao et al. in the context of dual antibody positivity [16]. Therefore, we speculate that the virus may destroy the blood–brain barrier and cause the leakage of MOG and NMDAR ags into the peripheral blood, and then T helper cells are activated, thus increasing the recruitment and activation of specific B cells for MOG and NMDAR ags, ultimately leading to the production of MOG abs and NMDAR abs, but the specific mechanism needs further research to confirm (Fig. 4). Distinctly, our case lacks infection. Consequently, even in the absence of an infection, the destruction of immune cells can potentially trigger the release of antigens. This implies that the damage to the nervous system caused by MOGAD itself may also potentially lead to the production of NMDAR abs. Currently, it has been clarified that there are cases of coexisting anti-NMDAR encephalitis and MOGAD, as well as the potential reasons for the formation of double antibody positivity. However, it is unclear whether the two antibodies synergistically or independently play a pathogenic role, or whether one of them is the main pathogenic agent. It is found that anti-NMDAR antibodies recognize and bind to the NMDAR GluN1 subunit, and mediate the entry of NMDAR from the postsynaptic membrane surface into the membrane and the degradation of Lysosome, resulting in the reduction of postsynaptic membrane surface receptors, the damage of synaptic signal transmission, and ultimately the death of neurons [39]. However, little is known about the pathogenic mechanism of anti-MOG antibodies, which may be related to their mediated cytotoxic effects attacking oligodendrocytes, triggering an inflammatory cascade reaction, leading to demyelination and neuronal death [40]. This study found that most of the patients with co-occurrence of NMDARE and MOGAD had CSF leukocytosis (see Supplementary file 1), which may indirectly provide a supporting basis for infection may lead to double positive results with abs, but the above pathogenesis needs to be confirmed by further research.

### When MNOS is suspected, NMDAR antibodies and MOG antibodies in serum and CSF can be detected at the same time after rigorous evaluation

In cases of double antibody positivity, NMDAR abs, and MOG abs can be detected sequentially or simultaneously (see Supplementary file 1). In the setting of overlap between anti-NMDAR encephalitis and MOG-associated demyelinating disease, titer does not appear to be significantly associated with symptom severity [9]. However, the antibody titer fluctuates with the change of disease, and the NMDAR ab and MOG ab titer may increase when the disease occurs or recurs, while the titers of NMDAR ab and MOG ab may decrease or even become negative when the disease improves, which is speculated to be related to disease recurrence, response to treatment, and prognosis [41, 42]. The serum potency of MOG abs usually declines gradually over time, or they may remain positive for years or become seronegative in the presence or absence of immunotherapy [4]. Patients who remain MOG positive are more likely to relapse [43], and a decrease in MOG-ab titers is associated with a uniphasic course [44]. An early reduction in CSF anti-NMDAR ab titers is associated with good results [45]. In this study, MOG-ab in serum and anti-NMDAR-ab titer in CSF were reduced after treatment, and the prognosis was favorable, which was consistent with previous results. Serum MOG abs are consistently low-positive in this study, so continued immunomodulatory therapy and antibody titers are necessary during follow-up. Given the low-positive MOG-ab titer, it has been suggested that its clinical relevance is yet to be determined and the possibility of a false-positive MOG-ab result cannot be ruled out, but we believe this is unlikely and since the present study was a low-positive serum MOG-IgG titer measured by a fixed-cell assay that possessed at least one of the supportive clinical or MRI features of the pending diagnosis of MOGAD, which excludes the diagnostic red flags and meets the diagnostic criteria established by the International MOGAD Study Group [4], but low MOG-ab positivity still needs to be interpreted with caution. In addition to this, the group emphasized the importance of serum testing for MOG-IgG in patients with suspected MOGAD, which is not recommended to be routinely tested in CSF. However, when patients with suspected MOGAD present with cortical encephalitis, simultaneous serum and cerebrospinal fluid testing should be considered from the outset [46]. In addition to this, MOG abs are sometimes undetectable in serum and detected in cerebrospinal fluid when MONS cases recur [47]. Therefore, we recommend simultaneous serum and cerebrospinal fluid testing for NMDAR abs and MOG abs when MNOS is suspected to minimize misdiagnosis and underdiagnosis, but this should be done after rigorous evaluation by clinical workers.

### The treatment for cases with coexisting dual antibodies is the same as for single-antibody diseases, and these patients can benefit even more from early immunotherapy

Despite coexisting MOG and NMDAR antibodies, initial management remains similar to that of antibody-positive patients alone, and immunotherapy is the primary treatment for patients with MNOS [28]. First-line immunotherapy includes various combinations of steroid therapy, IVIG, plasmapheresis, or above. Second-line therapies include rituximab and cyclophosphamide [28, 48]. At present, the treatment regimen and duration of biantibody-positive patients are inconclusive, and even though individual case reports show significant efficacy of conventional dose steroid therapy [28], it is generally believed that consistent with mono antibody disease, high-dose steroids or IVIG is required in the acute phase. Immune maintenance therapy is required after the treatment. These patients have shown a better response to steroids and IVIG in acute settings, a higher risk of recurrence, a better overall prognosis, and are most likely to benefit from long-term immunomodulatory therapy [49, 50], possibly due to positive MOG abs [28, 38]. This study is consistent with previous results, sensitive to steroids, and has a good prognosis. Seizures caused by these autoimmune antibodies are usually self-limited or require short-term antiseizure medication and long-term antiseizure medication is not needed in most patients [19, 20] (see Supplementary file 1). Some patients are prone to relapse during hormonal tapering [9, 12, 34], and failure to receive second-line and/or repeated first-line immunotherapy is associated with relapse [51]. Therefore, these patients may require longer immune maintenance therapy or second-line therapy and are more likely to benefit from starting treatment early. Therefore, the initiation and duration of immune-maintenance therapy and the time point and regimen of second-line therapy may be future directions for research.

In conclusion, our case serves as a reminder to clinicians that when patients present with atypical clinical manifestations such as seizures, consideration should be given to MNOS and conduct testing for various relevant autoantibodies (including MOG abs) and viruses in both serum and cerebrospinal fluid, as it is easy to misdiagnose the disease as other CNS diseases, such as viral meningoencephalitis. In addition to this, the syndrome exhibits high responsiveness to steroids, highlighting the critical importance of recognizing the clinical and neuroimaging features of this overlap syndrome for prompt diagnosis and treatment. Furthermore, it enriches the disease spectrum of MNOS.

### Supplementary Information

**Supplementary Material.**

## Data Availability

The datasets used and/or analyzed during the current study are available from the corresponding author on reasonable request.

## Abbreviations

NMDAR: N-methyl-D-aspartate receptor
NMDARE: Anti-N-methyl-D-aspartate receptor encephalitis
AE: Autoimmune encephalitis
abs: Antibodies
MOG: Myelin oligodendrocyte glycoprotein
MOGAD: MOG antibody-related diseases
ON: Optic neuritis
ADEM: Acute disseminated encephalomyelitis
FLAIR: T2-weighted fluid-attenuated inversion recovery
FLAMES: Cortical T2-weighted fluid-attenuated inversion recovery (FLAIR) -hyperintense lesions in anti-MOG-associated encephalitis with seizures
MNOS: Overlapping syndrome of MOGAD and NMDARE
CNS: Central nervous system
CSF: Cerebrospinal fluid
EEG: Electroencephalograph
MRI: Magnetic Resonance Imaging
ab: Antibody
Ags: Antigens
Ref: Reference
M: Male
F: Female
WBC: White blood cells
MRI: Magnetic resonance Imaging
Cx: Cortex
M.E: Meninges enhancement
n.a: Not applicable
MOG: Myelin oligodendrocyte glycoprotein
AEDs: Antiepileptic drugs
IV: Intravenous
St: Steroids
IVMP: Intravenous methylprednisolone
PE: Plasma exchange
RX: Rituximab
IVIG: Intravenous immunoglobulins
OP: Oral prednisone
HDMT: High dose methylprednisolone pulse therapy
AZA: Azathioprine
MMF: Mycophenolate mofetil
DSM: Dexamethasone
MP: Methylprednisolone
MT: Metho-trexate
R: Relapse
M: Monophasic
DSE: Demyelination syndrome episode
