# Supplementary material for: FLAMES overlaying anti-N-methyl-D-aspartate receptor encephalitis: a case report and literature review
Source: BMC Neurol. 2024 Apr 25;24:140. doi: 10.1186/s12883-024-03617-z (PMC11044310; doi:10.1186/s12883-024-03617-z)
Supplement: Supplementary file 1 — Supplementary Material. [file 12883_2024_3617_MOESM1_ESM.pdf]

Supplementary file 1

FLAMES overlaying anti-N-methyl-D-aspartate receptor encephalitis: a case report and literature review

*** Correspondence:** Lili Cui: e-mail: cuilili@gdmu.edu.cn; or Yusen Chen: email: chenyusen925@163.com

Table S1: The coexistence of MOG and NMDAR antibodies with seizures has been reported during the disease.

Ref	Age/sex	Initial symptoms and seizures	MRI abnormality		MOG-ab (CSF/serum)	NMDAR-ab (CSF/serum)	CSF Leukocytosis(+/-)	Therapy			courses	Ultimate outcomes
			Lesions of seizures and the thalamus involved	M.E				Immunotherapy	AEDs (+/-)	Anti-viral (+/-)		
Patient 1(3)	10/F	Bilateral optic neuritis in the 1st episode; Focal seizures in the 2nd and 4th episodes.	Right parietal cx in the 2nd onset; A new lesion in the right frontal lobe in the 4th onset.	(-)	(-)/(+) at one year later.	(+)/(-) in the 4th onset.	(+)	IVMP, OP, MT, IVIG, and received RX prophylactic therapy.	(+)	(+)	R	Recovery
Patient 2(7)	31/M	Fever, headache, and generalized seizure in the 1st episode.	The right temporal lobe, parietal, and occipital cx in the 1st onset.	(-)	(-)/ (+) in the 4th and 5th onset.	(+)/(-) in the 4th and 5th onset.	(+)	IV DSM, OP, MP, IVIG, AZA, MP retrobulbar injection, HMDT, MMF.	(-)	(+)	R	Symptoms relieved
Patient 3(8)	29/M	Seizures, dysarthria, and hemiparesis in the 1st episode.	Frontotemporal in the 1st onset.	(-)	(+)/(+)	(+)/(-)	(-)	St, IVIG.	(-)	(-)	R	Sequelae due to DSE
Patient 4(9)	19/M	Fever, headache, seizure, memory impairment, and unresponsiveness in the 1st onset.	Normal in the 1st onset; The left frontal lobe, basal ganglia, thalamus, and pons are in the 2nd onset.	(-)	(-)/(+) in the 2nd onset.	(+)/(-) in the 2nd onset.	(+)	DSM, IVIG, IVMP, OP, AZA.	(-)	(-)	R	Recovery
Patient 5(10)	20/F	Seizure, disorientation, neck stiffness, headache, and fever at the time of onset.	Bilateral cingulate gyrus and the medial region of the superior frontal gyrus, in the upper part of the corpus callosum in the 1st	(-)	(+)/(-) at 27 days after admission.	(+)/(-) at 27 days after admission.	(+)	St, IVIG, PE.	(+)	(-)	M	Recovery

Ref	Age/sex	Initial symptoms and seizures	MRI abnormality		MOG-ab (CSF/serum)	NMDAR-ab (CSF/serum)	CSF Leukocytosis(+/-)	Therapy			courses	Ultimate outcomes
			Lesions of seizures and the thalamus involved	M.E				Immunotherapy	AEDs (+/-)	Anti-viral (+/-)		
			onset.									
Patient 6(11)	12/M	Fever, headache, loss of consciousness, and generalized tonic seizure in the 1st episode.	Left frontotemporal parietal occipital lobes and right temporal-parietal lobes in the 1st onset.	(-)	(-)/(+) in the 2nd onset.	(+) /(+) in the 2nd onset.	(+)	IVIG, IVMP, RX.	(-)	(+)	R	Sequelae due to DSE
Patient 7(12)	38/M	Right facial numbness, diplopia, blurred vision, etc. in the 1st onset; Tonic-clonic seizure in the 3rd onset.	New serpentine lesions involving the bilateral frontal, temporal, parietal, and occipital Cx (Lace sign), and brainstem lesion in the 3rd onset.	(-)	(-)/(+) in the 3rd onset.	(+)/(+) in the 3rd onset.	(+)	IVMP, OP, IVIG, MMF.	(-)	(-)	R	Recovery
Patient 8(13)	3.4/M	Seizure, abnormal behavior, etc.	Normal.	(-)	(+)	(+)	(+)	St, IVIG, RX, and tocilizumab	(-)	(-)	N.a	Recovery
Patient 9(14)	25/M	Fever, headache, agitation, seizures, etc. in the 1st episode.	Normal in the 1st onset.	(-)	(-)/(+) in the 5th episode.	(+)/(-) in the 5th onset.	(+)	IVMP, IVIG, OP, MMF, RX.	(-)	(-)	R	Symptoms relieved
Patient 10(15)	27/M	Headache, aggressive behavior, tonic-clonic seizure, etc.	Middle cerebellar peduncles, midbrain, and some supratentorial lesions.	(-)	(+)/(-)	(+)/(-)	N.a	IVMP, RX.	(-)	(-)	M	Recovery
Patient 11(16)	37/M	Headache, left limb weakness at the time of onset; Tonic–clonic seizure at 7 days after admission.	The lesion in the medial aspect of the bilateral frontal lobe after a seizure.	(+)	(+)	(+)	(+)	OP.	(+)	(-)	M	Recovery
Our case	29/M	Seizure.	The right thalamus.	(+)	(+)/(+)	(+)/(+)	(+)	IVMP, OP.	(+)	(+)	M	Recovery

Ref= reference; M= male; F= female; CSF= cerebrospinal fluid; WBC= white blood cells; MRI= magnetic resonance Imaging; Cx= cortex; FLAIR= fluid-attenuated inversion recovery; M.E= meninges enhancement; ab= antibody; n.a= not applicable; NMDAR= N-methyl-D-aspartate receptor; MOG= Myelin oligodendrocyte glycoprotein; AEDs= antiepileptic drugs; IV= Intravenous; St= steroids; IVMP= Intravenous

methylprednisolone; PE= plasma exchange; RX= rituximab; IVIG=intravenous immunoglobulins; OP= oral prednisone; HDMT= High dose methylprednisolone pulse therapy; AZA= azathioprine; MMF= mycophenolate mofetil; DSM= dexamethasone; MP= methylprednisolone ;MT= metho-trexate; R= relapse; M=monophasic; DSE= demyelination syndrome episode.
